# Multiomics Identifies Potential Molecular Profiles Associated With Outcomes After BRAF-Targeted Therapy in Patients With BRAF V600E-Mutated Advanced Solid Tumors

**DOI:** 10.1200/PO.24.00266

**Published:** 2025-03-13

**Authors:** Martina Eriksen, Anne M. Hansen, Annelaura B. Nielsen, Filip Mundt, Matthias Mann, Ulrik Lassen, Lise B. Ahlborn, Martin Højgaard, Iben Spanggaard, Camilla Qvortrup, Christina W. Yde, Kristoffer S. Rohrberg

**Affiliations:** ^1^ Department of Oncology, Rigshospitalet, Copenhagen University Hospital, Copenhagen, Denmark; ^2^ Department of Genomic Medicine, Rigshospitalet, Copenhagen University Hospital, Copenhagen, Denmark; ^3^ Novo Nordisk Foundation Center for Protein Research, University of Copenhagen, Copenhagen, Denmark

## Abstract

**PURPOSE:**

It is a clinical challenge to select patients for BRAF-targeted therapy because of the lack of predictive biomarkers besides the BRAF V600E mutation. By analyzing the genome, transcriptome, and proteome, this study investigated the association between baseline molecular alterations and outcomes of BRAF-targeted therapy.

**PATIENTS AND METHODS:**

Fresh tumor tissue from patients enrolled in the Copenhagen Prospective Personalized Oncology study was collected and underwent comprehensive molecular profiling.

**RESULTS:**

*TP53* comutations were most frequently detected. Patients with a *TP53* wild-type tumor had a significantly longer median progression-free survival than those with *TP53* comutations (hazard ratio, 2.8 [95% CI, 1.13 to 7.08]; *P* = .02). RNAseq revealed a distinct gene expression signature for patients with long-term disease control (LDC), including hallmarks of cell cycle arrest and proliferation in the p53 pathway. The protein analysis demonstrated that ubiquitin-conjugating enzyme EK2 was significantly downregulated in patients with LDC.

**CONCLUSION:**

Using a multiomic approach, we identified molecular alterations associated with treatment outcomes. The potential of analyzing multiomic data is promising and should be prioritized in translational cancer research to uncover the full potential within precision oncology.

## INTRODUCTION

*BRAF* mutations are well known as oncogenic drivers in several cancers and are found in approximately 6% of all cases of metastatic cancer.^1^ Activating *BRAF* mutations lead to constitutive activation of the mitogen-activated protein kinase (MAPK) pathway resulting in uncontrolled cell proliferation, dedifferentiation, and cell survival.^2^ The most common *BRAF* mutation in human cancer is the BRAF V600E mutation.^3,4^

**CONTEXT**

**Key Objective**
Are there molecular alterations associated with outcomes to BRAF-targeted therapy in patients with BRAF V600E-mutated advanced solid tumors?
**Knowledge Generated**
Molecular alterations associated with outcomes to BRAF-targeted therapy were found using a multiomic approach analyzing RNA and protein expression in addition to DNA sequencing. Comutational status of the tumor suppressor gene *TP53*, and expression of the protein ubiquitin-conjugating enzyme EK2 were found to be associated with outcomes to the BRAF-targeted therapy.
**Relevance**
Knowledge on how molecular alterations affect outcomes to BRAF-targeted therapy in patients with BRAF V600E-mutated advanced solid tumors is an important tool that may help clinicians decide on the most appropriate therapy for the individual patient.

Although targeted therapies can be an effective strategy for treating cancer, the duration of responses to such therapies, for example, BRAF inhibitors (BRAFi), varies substantially and can be dependent on the origin of the primary tumor.^5-11^

Different molecular alterations have been proposed as predictive biomarkers of response to BRAF-targeted therapy besides the BRAF V600E mutation. For example, loss-of-function mutations in tumor suppressor genes have been shown to predict a lack of sensitivity to BRAF-targeted therapy and are negatively associated with survival outcomes in melanoma.^12-15^ By contrast, in colorectal cancer (CRC), loss-of-function mutations in specific tumor suppressor genes have been proposed to predict good response to BRAF-targeted therapy and improved survival.^16,17^ Generally, the impact of molecular coalterations regarding response and outcomes to BRAF-targeted therapy has yet to be fully elucidated.

Many proposed predictive biomarkers arise from data generated from DNA sequencing. In addition, more insights may be gained by analyzing transcriptomics and proteomics. Recently, we have enabled the extraction of material for mass spectrometry (MS)–based protein expression analyses from the same biopsy used for DNA and RNA sequencing.^18^ Proteins are the translated product of DNA alterations and critical molecules in all biological contexts.^19^ Additional insights, including a functional understanding of cancer signaling pathways, could potentially be gained with a multiomic approach.^20-22^

### Aim

This study aimed to investigate the association between baseline molecular characteristics and BRAF-targeted therapy outcomes in patients with BRAF V600E-mutated advanced solid tumors. We focused on the impact of comutations and on identifying molecular features associated with a long-term benefit of the therapy.

## PATIENTS AND METHODS

### Patient Cohort and BRAF-Targeted Therapy

This study comprises the subpopulation of patients with BRAF V600E-mutated solid tumors enrolled in the Copenhagen Prospective Personalized Oncology study (CoPPO).^23^ CoPPO was approved by the regional ethics committee (reference No.: 1300530) and all patients signed informed consent before inclusion.

The patients in the current study were all assigned to treatment with BRAF-targeted therapy between June 2016 and January 2021 at Copenhagen University Hospital, Rigshospitalet.

All were treated with a BRAFi combined with an anti-EGFR antibody or a MEK inhibitor or both.

Clinical data were collected from electronic medical records.

#### Response Evaluation

A baseline computed tomography (CT) scan was performed before the initiation of BRAF-targeted therapy, and response to treatment was evaluated with CT scans. The response was assessed according to RECIST version 1.1.^24^

#### Long-Term Disease Control and Survival Analysis

We defined long-term disease control (LDC) as duration of the BRAF-targeted therapy lasting 1 year or more before disease progression. In the analyses concerning patients with LDC, these patients are compared with the rest of the cohort in the study.

Overall survival (OS) was calculated from the start of therapy to death of any cause. Progression-free survival (PFS) was calculated from the start of therapy until progression, according to RECIST 1.1. The Cox proportional hazards model method was used to estimate the hazard ratio (HR) and 95% CI. The survival analyses were done in R Studio, version 4.1.1.

### Biological Material

In CoPPO, fresh tumor biopsies, stored in RNAlater, from a metastatic lesion, were obtained before initiating targeted therapy. The tumor tissue was used for molecular profiling, as described below. Peripheral blood samples were also obtained to analyze germline variants and circulating tumor DNA.

### Methods for Molecular Profiling of DNA and RNA

Tumor DNA and RNA were extracted from the tissue biopsies using the AllPrep kit from QIAGEN according to the manufacturer's instructions. Germline DNA was purified from blood using a Tecan liquid handler. DNA (tumor and germline) were processed to perform whole exome sequencing by standard protocols using 10-150 ng of DNA as input. Similarly, RNA was processed for sequencing using 10-200 ng of input. Library preparation was done with SureSelect Clinical Research Exome (Agilent, Santa Clara, CA) and TruSeq Stranded Total RNA Prep Gold (Illumina, San Diego, CA). Sequencing was performed on the Illumina NovaSeq6000 system. The raw data were mapped to the hg19/GRCh37 human reference genome using BWA-MEM software, version 0.7.12. Genome Analysis Toolkit best practices were followed when calling variants.

#### Mutational Analyses

DNA variants were analyzed using QIAGEN Clinical Insight Interpret. Somatic mutations were identified by subtracting the germline variants from the tumor variants. The American College of Medical Genetics and Genomics guideline^25^ was used to interpret the cancer-associated variants. In the current study, only variants classified as pathogenic or likely pathogenic were considered.

#### RNAseq Analyses

After mapping reads from the RNAseq analysis to the hg19/GRCh37 reference genome, a matrix of FeatureCounts was generated. Differentially expressed gene analysis was performed using DESeq2 (v.1.38.3) with FeatureCounts as input and based on the selected reference groups. As some patient samples contained elevated amounts of ribosomal RNA, all data sets were depleted of these genes using a gene list extracted from the RefSeq GTF database. Only genes with an adjusted *P* value of <.05 were used in downstream analyses.

The Gene Set Enrichment Analysis was computed using the R-package fgsea with a preranked gene list. Gene sets were restricted to a minimum size of 15 genes and a maximum of 400. The analysis was executed on gene sets from MSigDB^26^ using the H: hallmarks gene sets.

The heatmap was computed using the Pheatmap package on logarithm-transformed (rlog-transformed) data. Only the top 25 upregulated and downregulated genes were selected (log2FC, adjusted *P* < .05).

The volcano plot highlights the significantly upregulated and downregulated genes (*P* < .05).

The principal component analysis plot was generated with rlog-transformed data using the top 500 most variable genes.

### Methods for Mass Spectrometry–Based Proteomics

The complete method for the protein analyses is previously described.^18^ Briefly, the flowthrough containing proteins remained after the extraction of DNA and RNA using the QIAGEN AllPrep kit. In summary, ice-cold (–20°C) acetone (100%) was added to each sample in a total of four volumes. The mixture was inverted a few times and kept overnight at –20°C. The following day, a clearly visible cloudy precipitate had formed. After centrifugation for 10 minutes at 20,000 relative centrifugal force (RCF) at 4°C, the supernatant was removed, and the pellet was washed with approximately 500 µL of ice-cold (–20°C) acetone. The pellet was then air-dried for approximately 10 minutes, or until no liquid remained above it, without being overdried. Pellets were resuspended in 8M urea using sonication (Bioruptor Plus, with 15 cycles of 30 seconds on and 30 seconds off). For digestion, we added LysC at a 1:50 ratio (enzyme:protein; w/w) and incubated for 2 hours on a shaker at 800 rpm. The 8M urea was diluted four times down to 2M uwins 50 mM Tris-HCl (pH 8.0) buffer. Then we added trypsin at the same ratio and incubated overnight on a shaker at 800 rpm. The next day, digestion was stopped by acidification using formic acid (FA) to a final concentration of 1%.

To desalt, Sep-Pak cartridges tC18 (3 cc 200 mg) were used. Cartridges were initially conditioned with 2 mL of 100% acetonitrile (ACN), followed by 2 mL of 50% ACN/0.1% FA. Next, they were equilibrated with 4 × 2 mL of 0.1% TFA. The samples were centrifuged at 20,000 RCF for 10 minutes before loading onto the cartridge. The cartridge was washed/desalted with 3 × 2 mL of 0.1% trifluoroacetic acid (TFA), followed by washing/desalting with 2 mL of 1% FA to remove the TFA. The elution was performed using 2 × 1 mL of 50% ACN/0.1% FA. The eluate was then dried by speed-vacuuming at 45°C and resuspended in 50 µL of A*. Finally, the sample was ready for peptide concentration determination using the NanoDrop spectrophotometer (Thermo Scientific, Waltham, MA).

#### Liquid Chromatography and Tandem Mass Spectrometry Analyses

Two-hundred ng of digested peptides were loaded onto a disposable Evotip C18 trap column (Evosep Biosystems, Odense, Denmark). The Evotips were primed with 2-propanol and activated with 0.1% FA in ACN, followed by equilibration with 0.1% FA. Centrifugal force at 800 RCF for 1 minute was used to load the Evotips onto the trap column. Once loaded, the Evotips were washed with 0.1% FA, and 200 µL of 0.1% FA was added to the top of the disks to prevent drying. The Evosep One LC system (Evosep Biosystems) was used to transfer the mass spec–ready peptides to a mass spectrometer. The peptides were eluted and separated by a 15-cm PepSep column (150 µm inner diameter; Evosep) filled with 1.5 μm Reprosil-Pur C18 beads (Dr Maisch GmbH, Germany) using a standardized Evosep 44-minute gradient method. Buffer A (0.1% FA in MS–grade water) and buffer B (0.1% FA in ACN) were used during the process, and the column temperature was maintained at 60°C using a Sonation Nanospray Flex Column oven. The total sample throughput was 30 samples per day (30 SPDs).

The Trapped Ion Mobility Spectrometry (TIMS) quadrupole time-of-flight (TOF) mass spectrometer (Bruker timsTOF Pro or timsTOF SCP) was used in data-independent acquisition mode with TIMS. The liquid chromatographer was coupled to the hybrid TIMS quadrupole TOF mass spectrometer through a 15-cm Pepsep column. For data-independent acquisition parallel accumulation-serial fragmentation (diaPASEF), the method covered an m/z-range of 100-1,700 at MS1 to 400-1,200 at MS2. The method included two ion mobility windows per diaPASEF scan, with variable isolation window widths adjusted to precursor densities. Twenty-five diaPASEF scans were deployed at a throughput of 30 SPDs with a cycle time of 2.7 seconds. The ion mobility range was set to 1.6 Vs cm^–2^ and 0.6 Vs cm^–2^, and accumulation and ramp times were specified as 100 ms for all experiments. The collision energy was set from 20 eV (0.6 Vs cm^–2^) to 59 eV at 1/K0 (1.6 Vs cm^–2^).

DIA-NN 1.8.0.1 was used to search the proteomes, with a maximum mass accuracy tolerance of 15 ppm for MS1 and MS2 spectra. Trypsin/P was set as the protease, with up to one missed cleavage and a maximum of two variable modifications from a list of modifications. Precursor lengths ranged from 7 to 30 amino acids with a charge of 2 or 3. MBR was used for proteome analysis, and protein inference was turned off. Spectral library was generated on-the-fly, and proteotypic peptides were annotated using the “Reannotate” option in DIA-NN. Quantification mode was set to “Robust LC (high precision),” and the output was filtered on the basis of precursor *q*-value <1% and global protein *q*-value <1%.

#### Protein Analyses

DIAnn data output was processed using the Clinical Knowledge Graph.^27^ After removing proteins with a high number of missing values (maximum 30% missing values), missing values were imputed on the basis of a normal distribution (width = 0.3; downshift = 1.8). For the pairwise comparison of the low-response group to the LDC group, a two-sided unpaired *t*-test was performed. We applied a Benjamini-Hochberg false discovery rate of 5% to correct for multiple hypothesis testing. Proteins below this threshold are referred to as significant, whereas proteins with a *P* < .05 before multiple hypothesis correction are, in this study, referred to as protein candidates.

Pearson correlations between RNA and protein measurements were computed from a subset of the data where RNA and protein were measured for the same genes (n = 6,830) and samples (n = 20). RNA measures were the norm-transformed output from DESeq2, and protein measures were log2(label-free quantification [LFQ]) from DIAnn.

Volcano plot: red dots highlight the proteins with a *P* < .05 and a positive log2FC, while light blue dots represent the proteins with a *P* < .05 and a negative log2FC. The dark blue protein highlights the protein with a post hoc adjusted *P* < .05.

The heatmap was computed using the cluster-map package in python seaborn, selecting the top 25 upregulated and downregulated proteins on the basis of the log2FC among proteins with a *P* < .05 before correction in long-term versus short-term disease control. Patients were clustered on the basis of the protein profile (*z*-scored LFQ intensities).

The protein-protein correlation was computed on the basis of a data set of all proteins with a *P* < .05 before correction.

## RESULTS

### Included Patients

**FIG 1.**
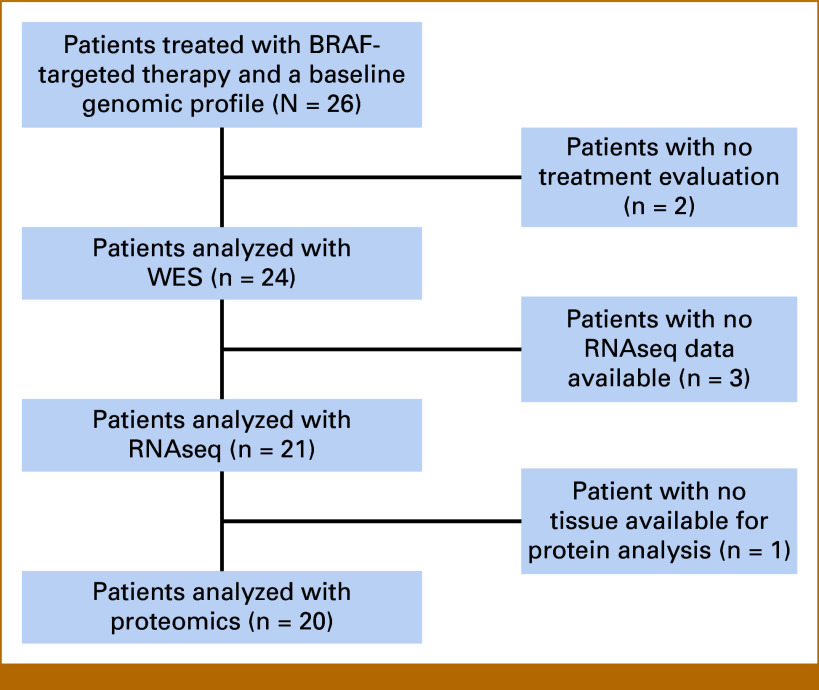
Flow diagram of the included patients. WES, whole exome sequencing.

Patients with a BRAF V600E-mutated, advanced solid tumor treated with BRAF-targeted therapy were identified. Figure 1 shows the flow diagram of the included patients.

Table 1 summarizes the demographics and clinical characteristics of the 24 included patients. Seven different histologies were represented. The BRAF-targeted therapy continued until progressive disease, and the best radiologic responses are reported in Table 1.

**TABLE 1.** Demographics and Clinical Characteristics of Patients (N = 24)

Characteristic	Number of Patients
Sex	
Male	14
Female	10
Age, years	
Median	62
Range	33-80
Primary tumors	
Colon cancer	15
NSCLC	3
NEC	2
MiNEN	1
Cholangiocarcinoma	1
Breast cancer	1
MM	1
BRAF-targeted therapy regimen	
Dabrafenib + trametinib	5
Dabrafenib + trametinib + panitumumab	4
Vemurafenib + panitumumab	4
Vemurafenib + panitumumab + irinotecan	3
Vemurafenib + cobimetinib	1
Encorafenib + binimetinib + panitumumab	3
Encorafenib + panitumumab	2
Encorafenib + binimetinib	1
Treatment duration, weeks	
Median	32
Range	8-230
Best radiologic response	
CR	1
PR	16
SD	7
PD	0

NOTE. Sex, median age, primary tumors, BRAF-targeted therapy regimens, duration of therapy (in rounded weeks) until progression, and the best radiologic response according to RECIST 1.1 are summarized.

Abbreviations: CR, complete response; MiNEN, mixed neuroendocrine non-neuroendocrine neoplasm; MM, malignant melanoma; NEC, neuroendocrine carcinoma; NSCLC, non–small cell lung cancer; PD, progressive disease; PR, partial response; SD, stable disease.

### Results of Mutational Analyses

**FIG 2.**
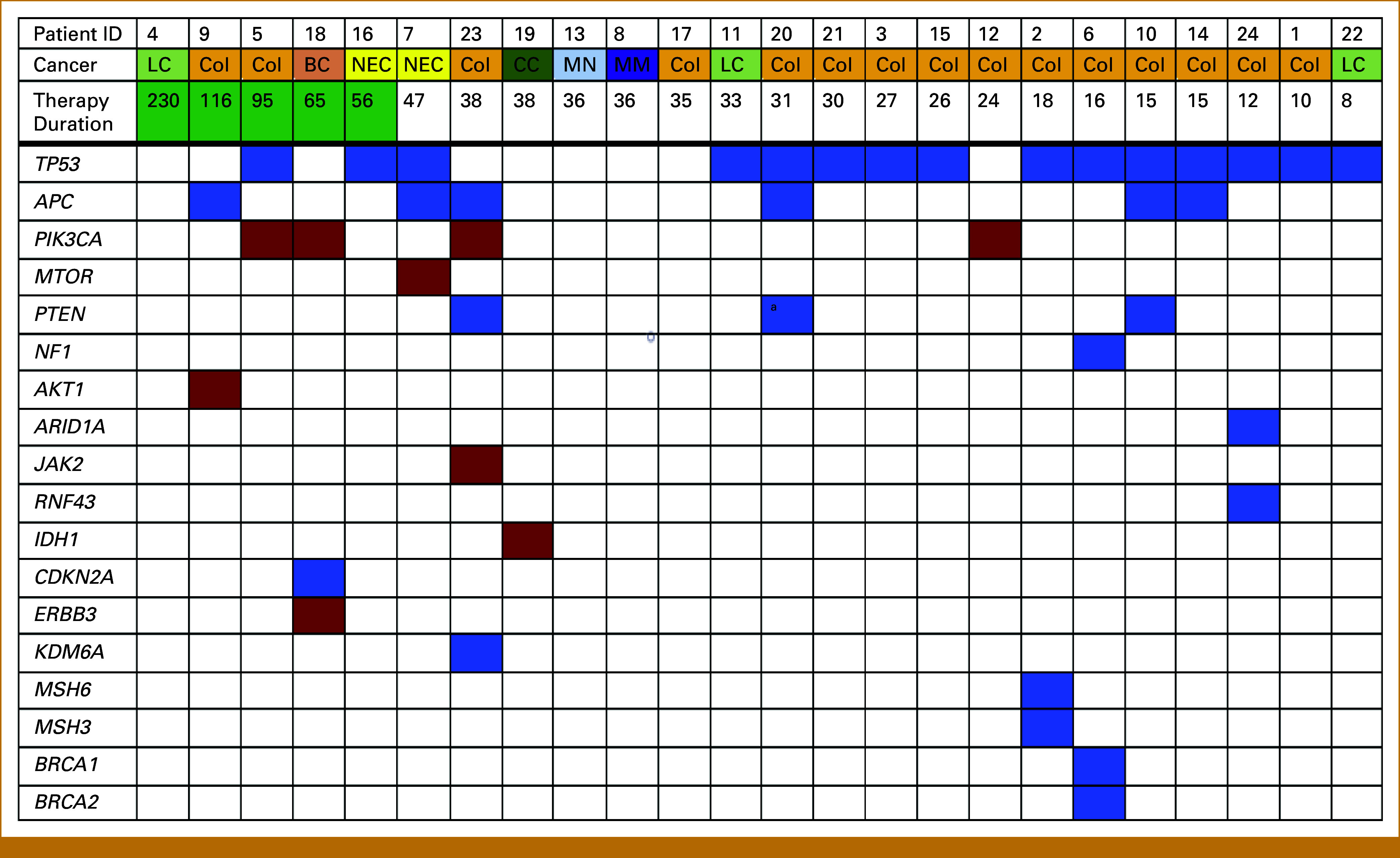
Baseline comutations in patients with BRAF V600E mutated solid tumors. Primary cancer, duration of response, and comutational status per patient are illustrated. The numbers at the top represent each patient, and primary cancer and therapy duration in weeks (rounded to the nearest whole week) is below. The bright green background in therapy duration marks patients with long-term disease control (more than 1 year). The colored boxes show that the patient had a mutation in the specific gene mentioned: red boxes represent gain-of-function mutations, and blue boxes loss-of-function mutations. ^a^In this patient, a *PTEN* gene fusion was detected. BC, breast cancer; CC, cholangiocarcinoma; Col, colon cancer; ID, identification; LC, lung cancer (all non–small cell lung cancer); MM, malignant melanoma; MN, mixed neuroendocrine non-neuroendocrine neoplasm; NEC, neuroendocrine carcinoma.

BRAF V600E mutation was detected in all included patients. Variants in the tumor suppressor gene *TP53*, resulting in loss of function, were the most frequently detected comutation (Fig 2). 62.5% (15/24) of the patients had at least one *TP53* comutation, with one patient having two comutations in *TP53*. For other variants found, consult Figure 2.

#### Impact of Comutations on Survival

**FIG 3.**
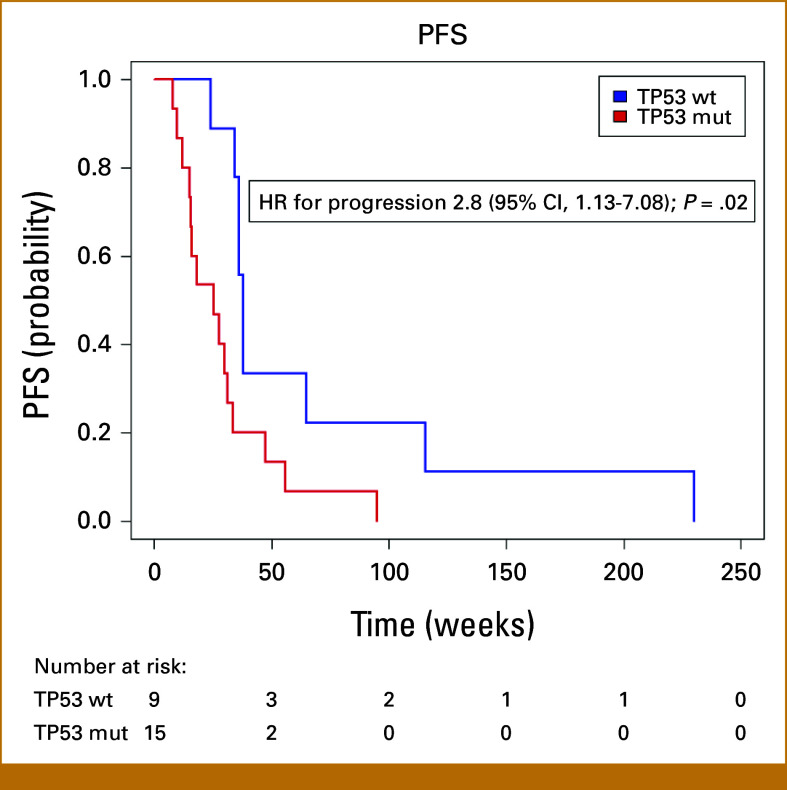
Kaplan-Meier plot of PFS in weeks in patients with BRAF V600E-mut solid tumors treated with BRAF-targeted therapy. The blue curve represents the subgroup of patients with no concurrent mutation in *TP53* at baseline. The red curve represents the patients with one or more *TP53* mutation(s) at baseline. There is a statistically significant difference in median PFS of 12.4 weeks between the two subgroups of patients (HR, 2.8 [95% CI, 1.13 to 7.08]; *P* = .02). HR, hazard ratio; mut, mutated; PFS, progression-free survival; wt, wild-type.

Patients with *TP53* wild-type (wt) tumors had a statistically significant longer median PFS of 12.4 weeks compared with patients with *TP53* comutated tumors (HR, 2.8 [95% CI, 1.13 to 7.08]; *P* = .02; Fig 3). The median OS was 78.6 weeks in the *TP53* wt subgroup of patients and 57.1 weeks in the *TP53*-mutated subgroup, but this difference was not statistically significant (HR, 1.3 [95% CI, 0.54 to 3.2]; *P* = .6). None of the other comutations were associated with survival.

### Results of RNAseq

**FIG 4.**
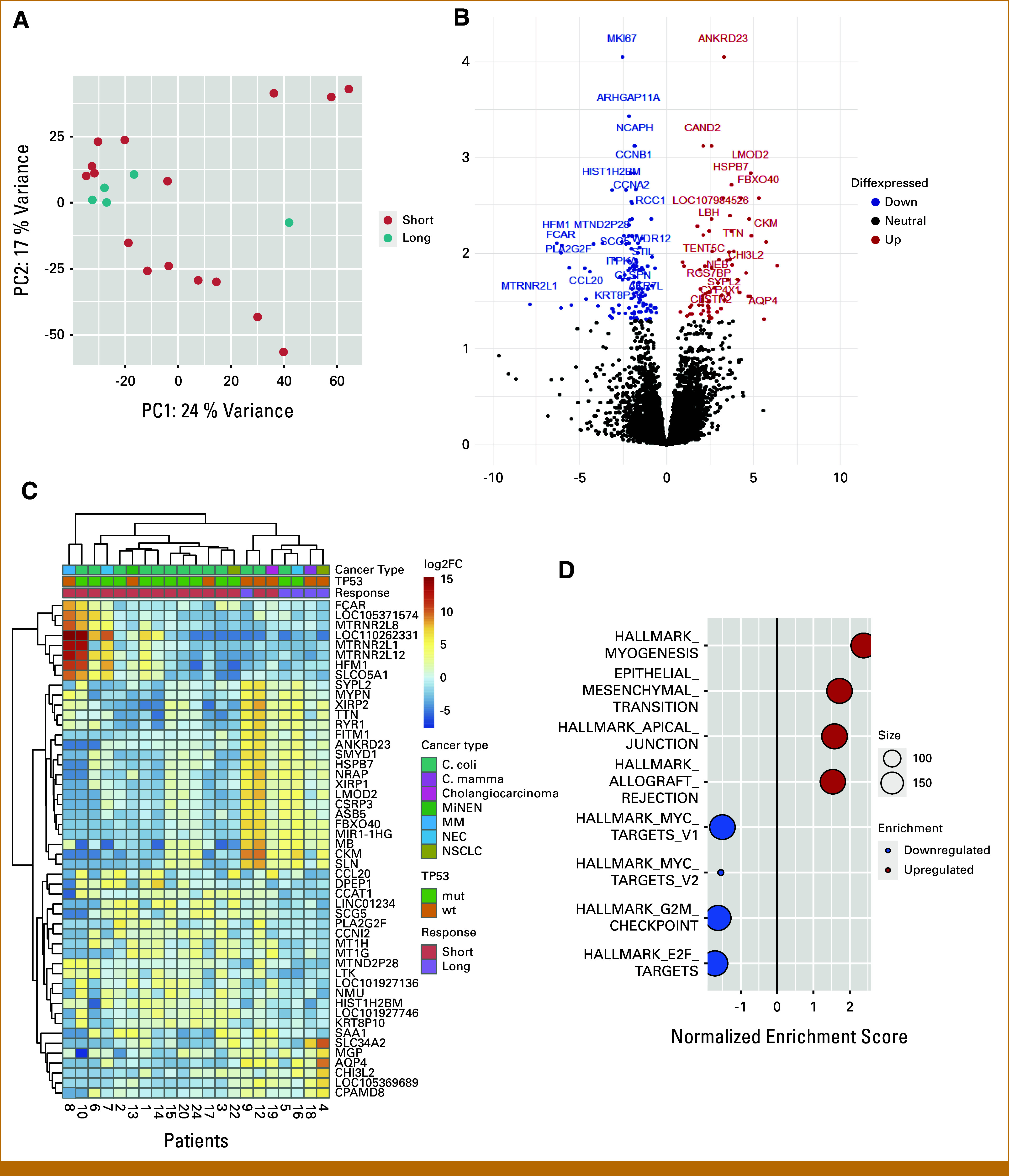
Gene expression profile in patients with LDC on the basis of RNAseq. (A) A PCA plot of patients with LDC (over 1 year) versus patients with shorter disease control (comprising rlog-transformed data using the top 500 most variable genes) showing that four of five patients clustered together. (B) A volcano plot of the significant (*P* < .05) differentially expressed genes in patients with LDC versus patients with shorter disease control. Seventy genes were significantly upregulated (red dots), and 124 genes were significantly downregulated (blue dots), *P* < .05, in patients with LDC. (C) A heatmap of the top 25 upregulated and downregulated genes (adjusted *P* < .05) with logarithmically transformed data. Patients were clustered on the basis of the deregulated genes, and a gene expression signature was indicated. Primary cancer, *TP53* mutational status, and duration of disease control are depicted at the top. (D) A GSEA plot illustrating upregulated and downregulated cancer hallmarks in patients with LDC versus shorter disease control. Pathways involved in cell cycle and proliferation were downregulated in patients with LDC. FC, fold change; GSEA, Gene Set Enrichment Analysis; LDC, long-term disease control; MiNEN, mixed neuroendocrine non-neuroendocrine neoplasm; MM, malignant melanoma; mut, mutated; NEC, neuroendocrine carcinoma; NSCLC, non–small cell lung cancer; PC, principal component; PCA, principal component analysis; wt, wild-type.

RNAseq data showed a distinct expression signature in patients with LDC (Fig 4). However, two patients with shorter disease control seemed to cluster with those with LDC (Fig 4C): one patient had colon cancer and disease control for 24 weeks until progression, and one had cholangiocarcinoma and disease control for 38 weeks. We observed a downregulation of cell cycle and proliferation hallmarks in the p53 pathway (Fig 4D).

### Results From Protein Analyses

On the basis of the expression profile from the RNAseq data in patients with LDC, we wanted to explore the protein expression in this subgroup.

**FIG 5.**
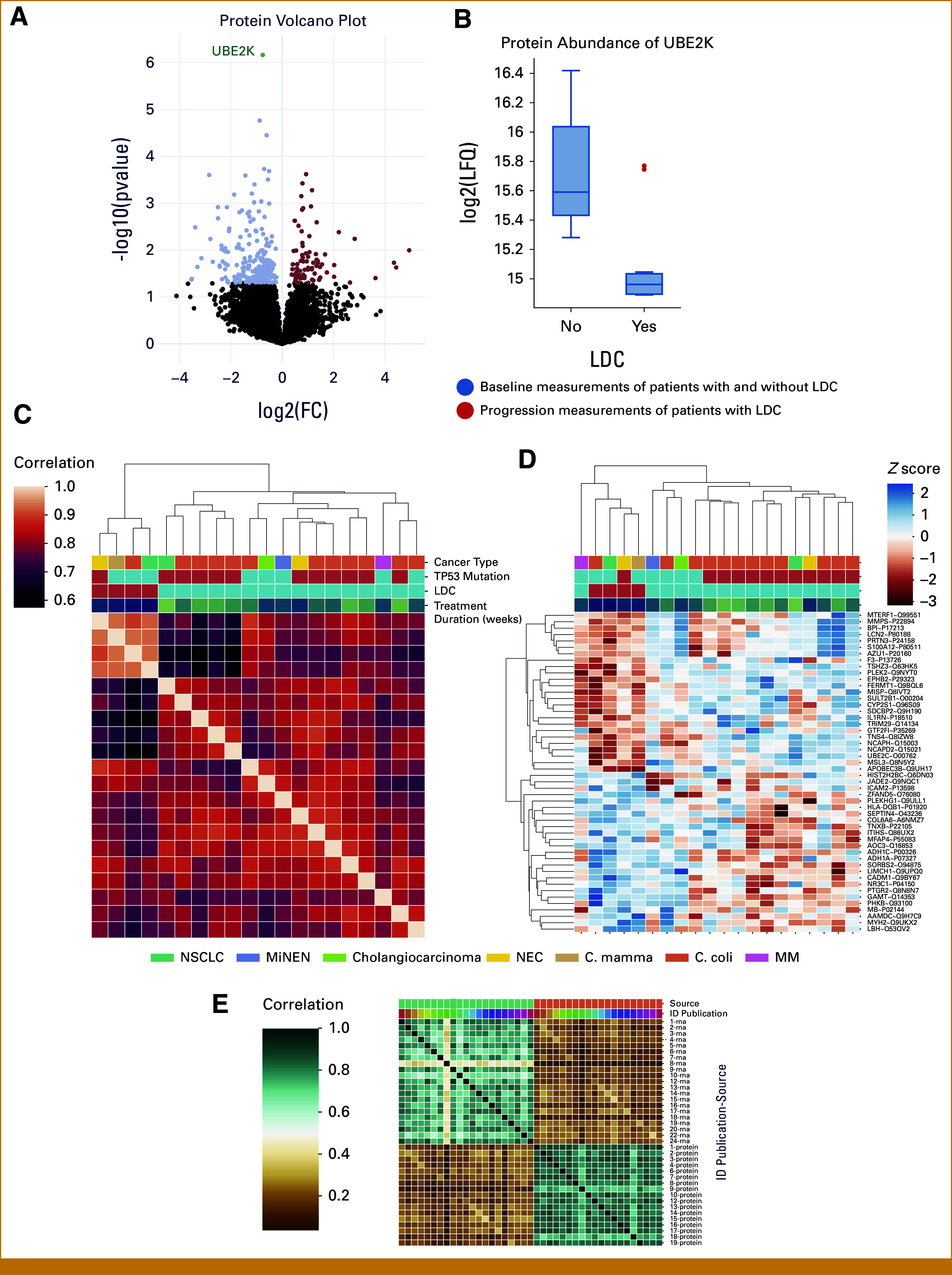
Protein expression profile in patients with LDC. (A) A volcano plot of the protein candidates in patients with LDC versus patients with shorter disease control. Upregulated proteins are red dots, and downregulated proteins are light blue dots. Significantly differently expressed proteins after multiple hypothesis corrections are illustrated with a green dot, and the name of the protein is written. (B) A box plot showing the abundance of UBE2K in patients with long-term and shorter-term disease control at baseline. Significant downregulation of UBE2K in patients with LDC is illustrated (with blue boxes). The two red dots show the abundance level of UBE2K at the time of progression in two (two samples were available from the progression time point for proteomic analysis out of four) patients with LDC. (C) The protein-protein correlation is shown. The correlation for each sample to all other samples in the data set was calculated and clustered according to correlation profiles. (D) A heatmap of the top 25 upregulated and downregulated protein candidates with a *P* < .05 before correction in long-term versus short-term disease control. Patients were clustered on the basis of the expression of proteins (zscored LFQ intensities). Primary cancer type, *TP53* mutational status (red box = mutated, blue box = wild-type), LDC (red box = yes, blue box = no), and duration of therapy (the darker the color, the longer the duration) are depicted at the top. (E) A correlation matrix between the measured RNA and protein samples. The correlation is calculated using the Pearson correlation. The turquoise boxes on the top represent the RNA samples, and the light orange boxes represent the protein samples. The color palette illustrates each patient in the second line from the top. The overall correlation is shown to be approximately 0.4. Twenty of the 21 patients from the RNAseq analyses were included in the protein analyses (Fig 1). The one patient with no tissue available for the protein analyses had LDC; therefore, four of the five patients with LDC were included in the protein analyses. FC, fold change; ID, identification; LDC, long-term disease control; LFQ, label-free quantification; MiNEN, mixed neuroendocrine non-neuroendocrine neoplasm; MM, malignant melanoma; NEC, neuroendocrine carcinoma; NSCLC, non–small cell lung cancer; UBE2K, ubiquitin-conjugating enzyme EK2.

Approximately 300 proteins were found to be differentially expressed (*P* < .05) among patients with LDC and the rest of the cohort (data not shown). After multiple hypothesis corrections, only one protein was significantly differentially expressed in the two groups: ubiquitin-conjugating enzyme EK2 (UBE2K),^28^ which was significantly downregulated in patients with LDC (Fig 5A). In two patients, we had paired samples available for the protein analyses. In those two patients, we could detect that the abundance of UBEK2 increased at the time of progression (Fig 5B).

The protein-protein correlation indicated similarities in protein expression in patients with LDC and revealed that patients cluster according to the duration of treatment, with a high, medium, and low cluster (Fig 5C). When clustering the patients on he basis of the top 25 upregulated and downregulated protein candidates, we identified protein expression profiles associated with LDC (Fig 5D).

Overall, the correlation between RNA and protein was low, around 0.4 (Fig 5E).

## DISCUSSION

In our study, using a multiomic approach, we identified potential predictive biomarkers of response to BRAF-targeted therapy in patients with BRAF V600E-mutated advanced solid tumors.

*TP53* is a tumor suppressor gene and is involved in the regulation of cell growth.^29^ *TP53* is essential for maintaining genomic stability and preventing oncogenic transformation,^30^ and is the most frequently mutated gene in human cancer. In our study, patients with tumors that were *TP53* wt had a significantly longer median PFS than patients with *TP53* comutations (HR, 2.8 [95% CI, 1.13 to 7.08]; *P* = .02); however, this did not translate into a statistically significant difference in survival. These findings align with an exploratory study in BRAF V600E-mutated melanoma.^14^

Other tumor suppressor genes have been associated with outcomes of BRAF-targeted therapy. For example, a cell-line study identified loss-of-function mutations in *NF1* (a negative regulator of RAS activity) in tumors that were intrinsically resistant to treatment with BRAFi.^12^ In our study, one patient with colon cancer had a comutation in *NF1*: this patient had disease control on BRAF-targeted therapy for 16 weeks before progression. Another biomarker recently proposed as predictive for response to BRAF-targeted therapy in BRAF V600E-mutated CRC is loss-of-function mutations in *RNF43*.^17^ Elez et al^17^ included 46 patients with metastatic CRC and found that 43% harbored a comutation in *RNF43*. In patients with microsatellite stable (MSS) disease, comutations in *RNF43* were associated with improved efficacy outcomes after BRAF-targeted therapy compared with patients with wt *RNF43* tumors.^17^ In our study, 13 patients had MSS colon cancer. We detected only one *RNF43* mutation (c.770dupG, p.E258*—a predicted loss-of-function mutation). This patient was treated with encorafenib and panitumumab, and achieved partial response as the best response. However, the duration of response was only 12 weeks in this patient. Another finding in the study by Elez et al^17^ is the suggestion of a cross-talk between the MAPK pathway and the WNT pathway that may play a role regarding the antitumor activity of BRAF-targeted therapy in patients with CRC. Another tumor suppressor gene involved in the WNT pathway is *APC*. *APC* is the most frequently mutated oncogenic driver in CRC.^31^ In a study analyzing tumors from 468 molecular unselected patients with CRC, *APC* was found to have a prognostic value in predicting survival.^32^ Of interest, tumors lacking any *APC* mutations were found to have an inferior prognosis than tumors harboring only one *APC* mutation. However, two *APC* mutations, alongside mutations in *KRAS* and *TP53*, exhibited the poorest prognosis of all.^32^ In our study, six patients had at least one *APC* mutation. We did not observe an impact of *APC* mutational status on survival. However, one patient had two *APC* mutations concurrent with a *TP53* mutation and this patient had an OS of 17 weeks. This is a very limited OS, one of the shortest in our overall cohort. As expected, we did not detect any comutations in *KRAS* in our study, as *KRAS* and BRAF V600E mutations are considered mutually exclusive.^33^

Achieving LDC is a clinically meaningful goal in the advanced cancer setting. In our study, we found a distinct gene expression signature and a downregulation of cell cycle and proliferation hallmarks in the p53 pathway in patients with LDC. Of the five patients with LDC, three patients had *TP53* wt tumors, and two patients had a *TP53* comutation. Therefore, we speculate whether this implies functional differences in p53 regardless of the mutational status of *TP53*.

We found one protein, UBE2K, to be significantly downregulated in patients with LDC. To our knowledge, UBE2K has not previously been associated with outcomes in *BRAF*-mutated cancer. UBE2K is a part of the family of enzymes involved in linking glycine residues of ubiquitin to specific lysine residues of target proteins. It is highly expressed in the brain and associated with the neurologic disorder Huntington's disease.^34,35^ In cancer, it has been found that the expression of UBE2K is increased in hepatocellular carcinoma (HCC), and recently it was shown that UBE2K promotes progression of HCC.^36^ UBE2K seems to play an essential role in the regulation of cancer cell cycles and may be a target for anticancer therapy.^36-39^ The potential role of UBE2K in BRAF V600E-mutated cancer needs further investigation.

The major limitation of the current study is the small sample size. Furthermore, the study did not include any control data. We included patients with different primary tumors in our study, which may contribute to heterogeneity of the population. However, we found UBE2K significantly downregulated in patients with LDC across four different *BRAF*-mutated primary tumors. Furthermore, the patients in our study represent a highly selected group of patients and may constitute another population than patients treated with BRAF-targeted therapy in daily routine practice.

In conclusion, in the surge of reaching the goal of precision oncology, it is key to be able to identify patients who will benefit the most from a specific therapy, as it seems to delay the development of acquired resistance, generally hindering a durable clinical benefit.^40-43^

We found that *TP53* mutational status was related to PFS. By RNAseq, we demonstrated that patients with LDC had downregulation in cell cycle and proliferation hallmarks in the p53 pathway. Proteomic data revealed that one protein, UBE2K, was significantly downregulated in patients with LDC across four different histologies. Although the expression of UBE2K is known to have a prognostic value in HCC, any potential role of UBE2K in *BRAF*-mutated cancer has not been established. The limited sample size in our study must be carefully considered, and therefore, any conclusions regarding UBE2K in *BRAF*-mutated cancer cannot be made.

The findings of our study need further validation, preferably in larger prospective patient cohorts. The potential implications of analyzing transcriptomics and proteomics, in addition to genomics, seem to provide valuable new insights and should be a prioritized focus for translational cancer research in the future.

## Data Availability

A data sharing statement provided by the authors is available with this article at DOI https://doi.org/10.1200/PO.24.00266. The data underlying this manuscript are sensitive in nature as they include human subject data, and therefore, the data cannot be made publicly available.
